# SIRT3 Enhances Mesenchymal Stem Cell Longevity and Differentiation

**DOI:** 10.1155/2017/5841716

**Published:** 2017-06-21

**Authors:** Ryan A. Denu

**Affiliations:** University of Wisconsin-Madison School of Medicine and Public Health, Madison, WI, USA

## Abstract

Mesenchymal stem cells (MSCs) are multipotent cells that are currently being investigated in a wide variety of clinical trials for their anti-inflammatory and immunomodulatory properties as well as their osteogenic and chondrogenic capabilities. However, there are considerable interdonor variability and heterogeneity of MSC populations, making it challenging to compare different products. Furthermore, proliferation, differentiation, and immunomodulation of MSCs decrease with aging and ex vivo expansion. The sirtuins have emerged as a class of protein deacylases involved in aging, oxidative stress, and metabolism. Sirtuin 3 (SIRT3) is the major mitochondrial deacetylase involved in reducing oxidative stress while preserving oxidative metabolism, and its levels have been shown to decrease with age. This study investigated the role of SIRT3 in MSC differentiation and aging. As MSCs were expanded ex vivo, SIRT3 levels decreased. In addition, SIRT3 depletion reduced MSC differentiation into adipocytes and osteoblasts. Furthermore, overexpression of SIRT3 in later-passage MSCs reduced aging-related senescence, reduced oxidative stress, and enhanced their ability to differentiate. These data suggest that overexpressing SIRT3 might represent a strategy to increase the quality and quantity of MSCs utilized for clinical applications.

## 1. Introduction

Mesenchymal stem/stromal cells (MSCs) are found in virtually every tissue in the body [1–3] and possess the ability to differentiate into adipocytes, osteoblasts, and chondrocytes. MSCs also suppress T and B cell proliferation [4], stimulate Tregs [5, 6], and change the immunophenotype of macrophages to a more immunosuppressive M2 phenotype [7]. Due to these immunomodulatory properties, MSCs are being studied for their potential use in inflammatory diseases and to heal injured tissue, such as graft-versus-host disease (GVHD), myocardial infarction, rheumatoid arthritis, Crohn's disease, type 1 diabetes mellitus, glomerulonephritis, liver disease, osteoarthritis, and multiple sclerosis [8–14].

One major challenge facing clinical utilization of MSCs is that they must be expanded ex vivo in order to be utilized in the clinic. However, as MSCs are expanded, they undergo replicative senescence [15–17] and demonstrate morphological abnormalities, enlargement [18, 19], more podia and actin stress fibers [18, 20], less expression of specific surface markers, and decreased proliferation and differentiation [21–28]. Furthermore, this replicative senescence also compromises their immunomodulatory and differentiation functions and possibly their clinical activity against GVHD and other inflammatory pathologies [29, 30]. These effects are thought to be at least partially due to higher ROS and oxidative stress in aged stem cells [31, 32]. Importantly, it remains unclear whether this physiological stress can be prevented or reversed.

Sirtuins are a class of NAD^+^-dependent protein deacylases with important implications in aging [33]. Humans have 7 sirtuins (SIRT1–7) with distinct subcellular localizations and functions. SIRT3 is the major mitochondrial deacetylase that decreases mitochondrial reactive oxygen species (ROS) and promotes efficient oxidative metabolism [34–37]. SIRT3 has been shown to slow age-associated decline of function and senescence in multiple cell types, and overexpression in hematopoietic stem cells (HSCs) from older donors enhances their longevity and differentiation [31]. Therefore, this study tested the hypothesis that SIRT3 plays an important role in MSC biology and could be manipulated to enhance MSC longevity. Herein, data demonstrate that SIRT3 decreases in MSCs with in vitro passaging, that knockdown of SIRT3 accelerates aging and inhibits differentiation into adipocytes and osteoblasts, and that overexpression of SIRT3 in later-passage MSCs restores their differentiation capacity and reduces oxidative stress. These results have implications for the ex vivo expansion of MSCs for clinical utilization.

## 2. Materials and Methods

### 2.1. Isolation and Culture of Mesenchymal Stem Cells

Human bone marrow MSCs were kindly provided by Peiman Hematti. They were derived and cultured, and their immunophenotype was assessed as previously described [3, 7].

### 2.2. SIRT3 Knockdown and Overexpression

Retrovirus expressing shRNA against SIRT3 (5′-GCGCCTATCAGTACACAAT-3′ for SIRT3-KD1, 5′-CTCCTCTGTTGCCTTGGTA-3′ for SIRT3-KD2) or scrambled (5′-CAACAAGATGAAGAGCACC-3′) was made by cotransfecting pSUPERIOR.retro.puro (Oligoengine) construct containing the above shRNA sequences with pVSV-G retroviral packaging vector into Phoenix cells (ATCC). Retrovirus was collected from supernatant of Phoenix cells and used to infect MSCs, which were subsequently selected with 0.5 *μ*g/mL puromycin for 5 days. Puromycin was removed prior to differentiation experiments. For SIRT3 overexpression, SIRT3 cDNA was cloned into pcDNA3.1 vector [34] and transfected with Fugene (Promega). Efficiencies of knockdown and overexpression were assessed by western blots.

### 2.3. Western Blotting

Cells were collected and incubated for 30 minutes on ice in lysis buffer (50 mm HEPES, pH 7.5, 100 mm NaCl, 0.5% Nonidet P-40, and 10% glycerol) containing phosphatase inhibitors (10 mm sodium pyrophosphate, 5 mm *β*-glycerol phosphate, 50 mm NaF, and 0.3 mm Na_3_VO_4_), 1 mm PMSF, 1X protease inhibitor mixture (Thermo Scientific), and 1 mm dithiothreitol. Proteins were separated by SDS-PAGE using 10% acrylamide gels, transferred to the Immobilon PVDF membrane (Millipore), and blocked for 1 hour in 5% bovine serum albumin (BSA) in Tris-buffered saline (TBS), pH 7.4. Membranes were incubated overnight at 4°C with primary antibodies diluted in TBST (TBS + 0.1% Tween 20) + 5% BSA, washed three times with TBST, and incubated for 1 hour at room temperature with secondary antibody conjugated to horseradish peroxidase (Jackson) in TBST + 5% BSA. Membranes were washed and developed with luminol/peroxide (Millipore) and visualized with film. Primary antibodies used in this study include SIRT3 (Cell Signaling Technologies, C73E3, 1 : 1000) and tubulin (Abcam, ab4074, 1 : 5000).

### 2.4. Flow Cytometry

MSCs were resuspended in 100 *μ*L PBS and incubated with 10 *μ*L of each antibody for 30 minutes at 4°C in the dark. The following antibodies were used: CD14 FITC, CD19 PE, CD34 APC, CD45 PE, CD73 PE, CD90 PE, CD105 PE, and HLA-DR FITC (BD Biosciences). As a control, cells were stained with the appropriate isotype antibodies. Cells were then washed with buffer and analyzed using an Accuri C6 Flow Cytometer. FlowJo software (Tree Star) was used to analyze the data. For measurement of ROS, 5 × 10^5^ cells were plated in a 24-well plate and cultured for 2 days. Cellular ROS was measured by staining with dihydroethidium (DHE, Sigma), as previously described [34, 38]. A total of 50,000 events were acquired using an Accuri C6 flow cytometer (Accuri) equipped with multicolor analysis, and data were analyzed with FlowJo. Unstained cells, cells treated with H_2_O_2_, and cells treated with the ROS scavenger N-acetyl-cysteine (NAC, Sigma) all served as controls.

### 2.5. Senescence Assay

Senescence was assessed using the Senescence *β*-Galactosidase Staining Kit (Cell Signaling) in 24-well plates. To assess the impact of SIRT3 expression on senescence, 5 × 10^4^ cells were plated in a 6-well plate following knockdown or overexpression of SIRT3 (as described above) and passaged for 14 days. After this time, 2 × 10^4^ of these cells were plated per well in a 24-well plate, fixed, and stained per the manufacturer's instructions. To assess the impact of ex vivo expansion on senescence, MSCs were continually passaged in T25 plates, and 2 × 10^4^ cells from each passage were seeded per well in 24-well plates for fixation and staining. 24 hour exposure to 4 *μ*M doxorubicin was used as a positive control for senescence.

## 3. MSC Differentiation

MSCs were differentiated as previously described [39]. Briefly, cells were plated in a 24-well plate and grown to near confluency. Adipogenic and osteogenic differentiation media (Miltenyi Biotech) were added and changed every 3 days for a total of 21 days. Adipocyte lipid droplets were detected by oil red O staining (Sigma). Osteoblast calcification was detected by alizarin red S staining (Sigma). Oil red O and alizarin red S were quantified by drying the wells, eluting with isopropanol, and reading absorbance at 500 nm and 520 nm, respectively.

### 3.1. Quantitative Reverse Transcription Polymerase Chain Reaction (qRT-PCR)

RNA was isolated from cells using the RNeasy Micro Kit (Qiagen) and converted to cDNA using the QuantiTect Reverse Transcription Kit (Qiagen). Quantitation was performed using Power SYBR green master mix (Applied Biosystems) on a StepOnePlus instrument (Applied Biosystems). Sirtuin 1–7 mRNA levels were normalized to three housekeeping genes (*RRN18S*, *GAPDH*, and *ACTB*). Primers were synthesized by the University of Wisconsin-Madison Biotechnology Center, and sequences can be found in Supplemental Table 1 available online at https://doi.org/10.1155/2017/5841716.

### 3.2. Statistical Analysis

Statistical differences between experimental groups were analyzed using GraphPad Prism 6 and Microsoft Excel 2011 software using *t*-tests and ANOVA to assess *P* values. Indications are made in the figures for *P*  values < 0.05 (∗).

## 4. Results

### 4.1. SIRT3 Expression Decreases with MSC Aging and Increases with Differentiation

**Figure 1:**
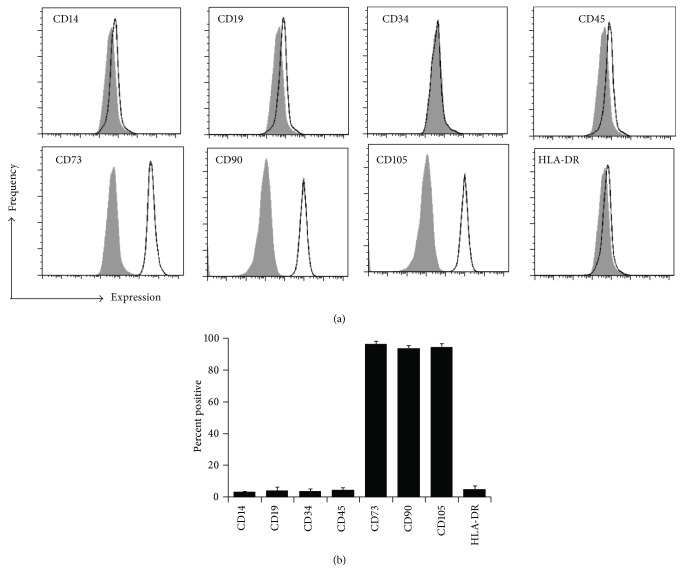
Immunophenotype of MSCs. (a) MSCs were isolated from bone marrow aspirates of 3 healthy donors. Representative flow cytometry histograms are shown. The black line indicates cells stained with the indicated antibody, while the gray histogram indicates cells stained with the isotype control. Passage 3 cells were used. (b) A bar graph demonstrating the average percent of positive cells for 3 MSC strains ± SEM.

**Figure 2:**
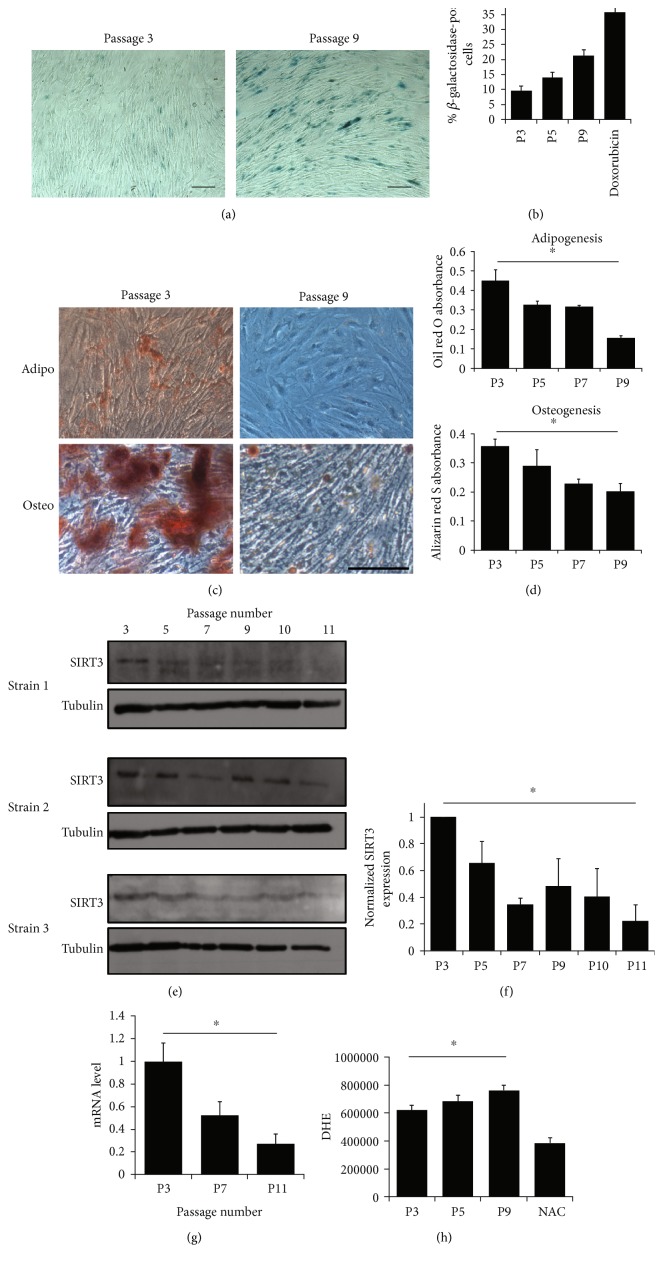
Differentiation capability and SIRT3 expression decrease with passaging. (a) To assess senescence, cells were assayed for *β*-galactosidase activity. Representative brightfield images of *β*-galactosidase staining in early- (passage 3) and late- (passage 9) passage MSCs. Scale bar = 100 *μ*m. (b) The percentage of cells stained in blue (indicating they are senescent) was assessed in 3 technical replicates from one MSC strain. 24-hour treatment of passage 5 MSCs with 4 *μ*M doxorubicin was used as a positive control for senescence. (c) Representative images of early- and late-passage MSCs after 21 days of treatment with appropriate differentiation media. Adipocytes were stained with oil red O, and osteoblasts were stained with alizarin red S. Scale bar = 100 *μ*m. (d) Oil red O and alizarin red S were eluted with isopropanol; then, absorbance was read at 500 nm or 520 nm, respectively. Bars represent means ± SEM in 3 technical replicates from one MSC strain. (e) SIRT3 expression was assessed in 3 different MSC strains after passaging for 11 passages. Alpha tubulin was used as a loading control. (f) Protein level from western blots in panel (e) was quantified using ImageJ and normalized to the SIRT3 level in passage 3 cells in each strain. Bars represent means ± SEM for the 3 MSC strains. (g) SIRT3 mRNA was measured by qRT-PCR. Cycle threshold (C_T_) values were normalized to the combination of 3 housekeeping genes (*RRN18S*, *GAPDH*, and *ACTB*). Relative mRNA values were calculated using 2^−ΔCT^, which were then normalized to passage 3 (P3) values. Bars represent means ± SEM for 3 MSC strains. (h) ROS were assessed by staining with DHE and analyzing by flow cytometry. N-Acetyl cysteine (NAC) scavenges ROS and was used as a control. Bars represent average DHE values (median channel fluorescence from FL3) ± SD from 3 technical replicates from one MSC strain. ^∗^*P* < 0.05 from one-way ANOVA tests.

Loss of SIRT3 is associated with aging-related phenotypes and pathologies, and its expression decreases with age [31, 40]. Therefore, the hypothesis was that SIRT3 expression decreases as MSCs are expanded in vitro. MSC strains were isolated from the bone marrow of three different donors and the expression of ISCT criteria-specified markers were assessed by flow cytometry at passage 3. All MSC strains used were positive for CD73, CD90, and CD105 and negative for CD14, CD19, CD34, CD45, and HLA-DR (Figure 1()). MSCs were passaged through 12 passages, and cells were collected every few passages and utilized for differentiation analyses. As MSCs were expanded in vitro, they became more senescent, as demonstrated by increased *β*-galactosidase activity (Figures 2(a) and 2(b)). Furthermore, their ability to differentiate into adipocytes and osteoblasts decreased as they were passaged (Figures 2(c) and 2(d)). In addition, SIRT3 expression decreased as MSCs were passaged, as demonstrated by quantification of protein expression (Figures 2(e) and 2(f)) and gene expression (Figure 2(g)). This correlated with an increase in ROS as MSC passage number increased (Figure 2(h)). The gene expression of the other 6 sirtuins was also assessed by qRT-PCR as MSC passage number increases (Supplemental Figure 1). In general, there was a trend toward decreased sirtuin expression as MSCs were expanded in vitro. This trend was most pronounced for SIRT1, SIRT3, and SIRT6.

**Figure 3:**
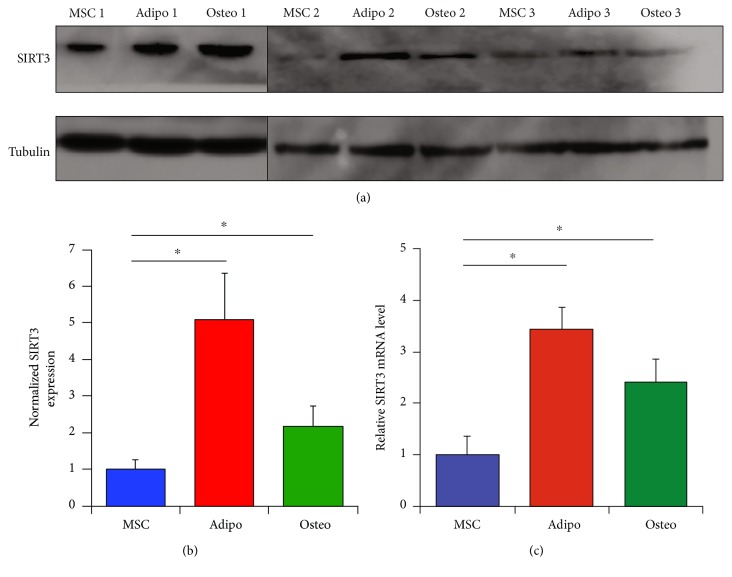
SIRT3 increases with MSC differentiation. (a) Western blotting demonstrating SIRT3 expression as 3 strains of passage 3 MSCs are differentiated into adipocytes and osteoblasts using appropriate induction media for 21 days. (b) Quantification of protein expression in western blots from panel (a). Expression was quantified using ImageJ and normalized to the expression of untreated MSCs for each of the 3 strains. (c) Quantitative RT-PCR demonstrating SIRT3 mRNA expression in 3 strains of MSCs after 21 days of exposure to appropriate differentiation media. Cycle threshold (C_T_) values were normalized to the combination of 3 housekeeping genes (*RRN18S*, *GAPDH*, and *ACTB*). Bars represent means ± SEM of 2^−ΔCT^ values. ^∗^*P* < 0.05 from two-sided *t*-tests comparing adipocytes and osteoblasts to undifferentiated MSCs.

Next, the expression levels of SIRT3 as MSCs differentiated into adipocytes and osteoblasts were examined. SIRT3 protein and mRNA expression were elevated in adipocytes and osteoblasts compared to the MSCs from which they were derived (Figures 3(a), 3(b), and 3(c)). In addition, the gene expression of the other 6 sirtuins was also assessed as MSCs differentiated into adipocytes and osteoblasts (Supplemental Figure 2). Adipocytes demonstrated a significant increase in SIRT1–6 gene expression, while osteoblasts demonstrated a significant increase in SIRT3 and SIRT5 gene expression.

### 4.2. SIRT3 Enhances MSC Differentiation

**Figure 4:**
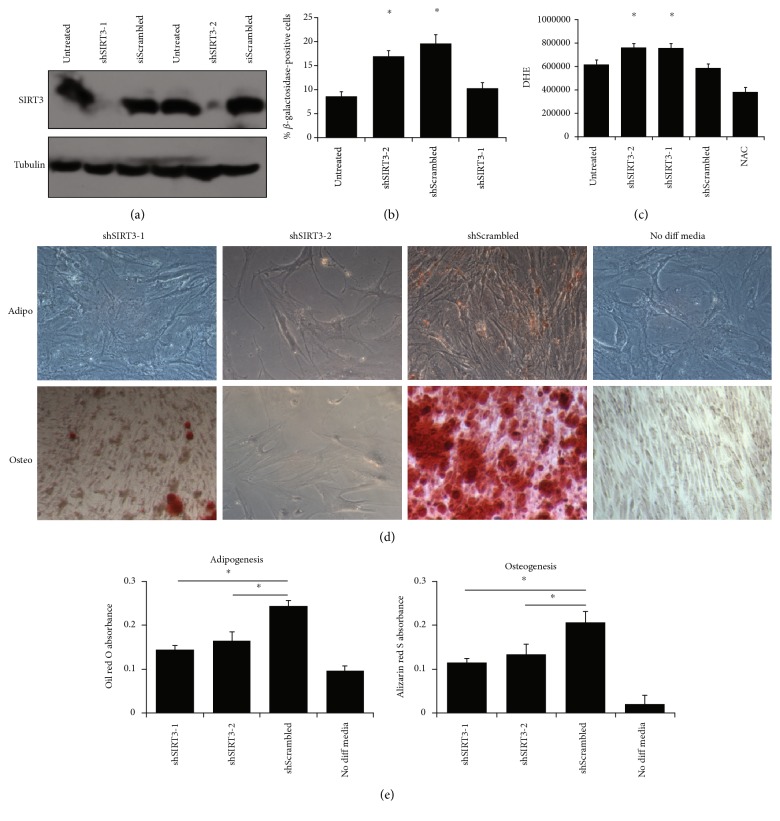
SIRT3 is required for MSC differentiation. (a) Passage 3 MSCs were transduced with retrovirus expressing shRNA against SIRT3 or scrambled control. Two different SIRT3 shRNA sequences were used. Western blotting was used to demonstrate efficient depletion of SIRT3. (b) To assess senescence, *β*-galactosidase activity assay was performed. Bars represent means ± SEM for the 3 MSC strains. ^∗^*P* < 0.05 from two-sided *t*-tests comparing shSIRT3-1 and shSIRT3-2 to both untreated and shScrambled conditions. (c) To assess ROS, MSCs were stained with DHE, which was detected by flow cytometry. Bars represent average DHE values (median channel fluorescence from FL3) ± SEM from the 3 MSC strains. “Untreated” refers to wild-type MSCs that were not virally transduced. ^∗^*P* < 0.05 from two-sided *t*-tests comparing shSIRT3-1 and shSIRT3-2 to both untreated and shScrambled conditions. (d) MSCs were differentiated for 21 days with appropriate adipogenic or osteogenic induction media. Adipocytes were stained with oil red O, and osteoblasts were stained with alizarin red S. Representative images are shown. Wild-type MSCs not treated with differentiation media but still stained with either oil red O or alizarin red S served as controls. Scale bar = 100 *μ*m. (e) Isopropanol was used to elute the oil red O and alizarin red S, and absorbance was read at 500 nm or 520 nm, respectively. Bars represent means ± SEM for 3 MSC strains. ^∗^*P* < 0.05 from two-sided *t*-tests comparing shSIRT3-1 and shSIRT3-2 to shScrambled.

To test the hypothesis that SIRT3 is required for MSC differentiation, SIRT3 was depleted in MSCs using two different shRNAs (Figure 4(a)), allowing for stable expression after many rounds of division and differentiation. SIRT3 knockdown caused an increase in senescence (Figure 4(b)) and ROS levels (Figure 4(c)). Furthermore, SIRT3 knockdown reduced the ability of MSCs to differentiate into adipocytes and osteoblasts. This was visualized by microscopy and quantified spectrophotometrically after staining with oil red O for adipocytes and alizarin red S for osteoblasts (Figures 4(d) and 4(e)). These data suggest that SIRT3 is required for efficient differentiation of MSCs into adipocytes and osteoblasts.

### 4.3. Overexpression of SIRT3 Restores Differentiation Capacity of Aged MSCs

**Figure 5:**
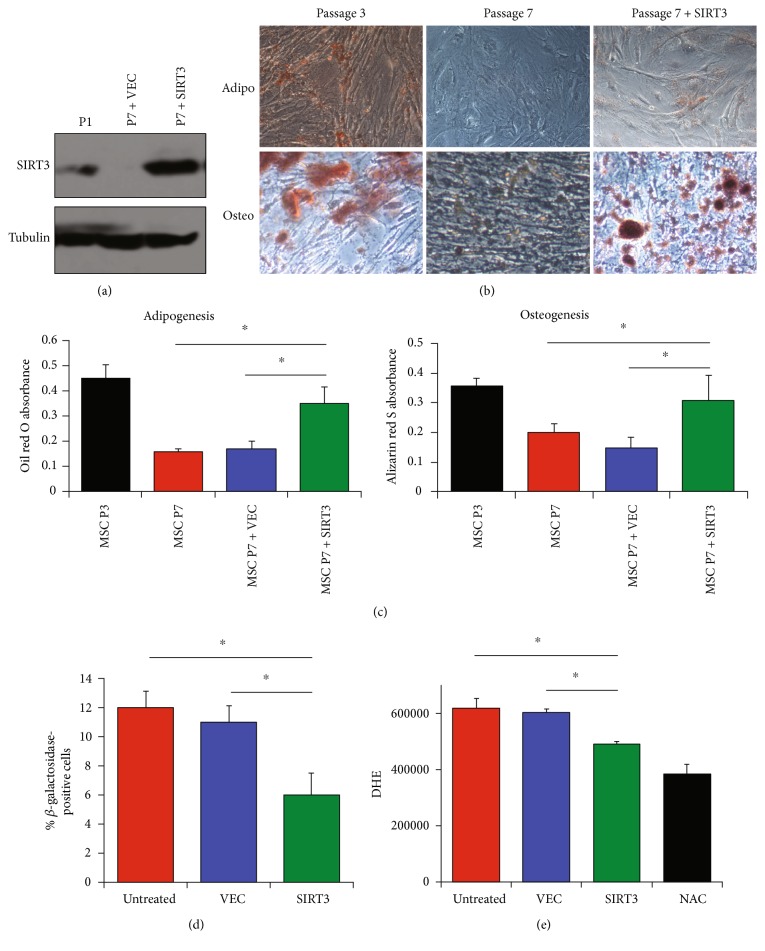
SIRT3 overexpression restores differentiation and reduces oxidative stress in aged MSCs. (a) SIRT3 was overexpressed in later-passage (passage 7) MSCs compared to untreated later-passage MSCs and early-passage (passage 1) MSCs and subsequently assessed by western blotting. VEC: empty vector control. Alpha tubulin served as a loading control. (b) Differentiation was assessed 21 days after the treatment with appropriate induction media. Adipocytes were stained with oil red O, and osteoblasts were stained with alizarin red S. Scale bar = 100 *μ*m. (c) Oil red O and alizarin red S were eluted with isopropanol; then, absorbance was read at 500 nm or 520 nm, respectively. (d) Senescence was assessed by quantifying the percentage of cells with *β*-galactosidase activity. Bars represent means ± SEM for the 3 MSC strains for 3 technical replicates of 1 MSC strain. “Untreated” cells were not transfected. (e) MSCs were stained with DHE, which was detected by flow cytometry. Bars represent average DHE values (median channel fluorescence from FL3) ± SEM from the 3 MSC strains. Cells treated with NAC, an ROS scavenger, served as a negative control. ^∗^*P* < 0.05 from two-sided *t*-tests.

The next question asked was whether or not increasing SIRT3 expression in older MSCs would reverse the effects of aging, restore the differentiation capacity, and reduce oxidative stress. To test this, SIRT3 was overexpressed in later-passage MSCs compared to the same later-passage MSCs treated with empty vector (Figure 5(a)) and to MSCs of the same strain from earlier passages. These cells were then induced to differentiate with proper differentiation media. Interestingly, SIRT3 overexpression in later-passage MSCs restored differentiation capacity (Figures 5(b) and 5(c)), reduced senescence (Figure 5(d)), and reduced ROS (Figure 5(e)) to approximately the same level as the early-passage MSCs. These results suggest that restoring SIRT3 expression in later-passage MSCs can help restore aging-associated deficits.

## 5. Discussion

Heretofore, sirtuins have not been well studied in MSCs. This investigation found that SIRT3 expression decreases as MSCs age and are passaged but increases as MSCs differentiate into adipocytes and osteoblasts. Depletion of SIRT3 reduced the ability of MSCs to differentiate into adipocytes and osteoblasts. Furthermore, overexpressing SIRT3 in later-passage MSCs reduced their oxidative stress and senescence and restored their differentiation capacity. These findings suggest that manipulating SIRT3 levels in MSCs may improve their ex vivo expansion.

MSCs have immense potential for tissue engineering and cell therapy. MSCs are currently in many ongoing clinical trials for a wide range of inflammatory diseases because of their ability to repair and inhibit inflammation. However, a number of setbacks have prevented MSCs from reaching their full clinical potential, as the biggest clinical trial involving MSCs to date failed to significantly reduce GVHD incidence and mortality [41]. First, MSCs are rare cells in situ and must be expanded ex vivo in order to be utilized in patients. However, MSCs undergo replicative senescence and experience attenuation of their immunomodulatory and differentiation functions when they are continually passaged, which may reduce their clinical utility against inflammatory diseases [15–17, 19, 29, 30, 42]. An additional reason for MSCs not reaching their full clinical potential is that MSC cultures are generally very heterogeneous, with cells at different stages of differentiation potential. Therefore, there is a great need (1) to find markers that identify MSCs with greater immunomodulatory and differentiation potential and (2) to develop ways to modify MSCs to make them more efficacious. Studies are trying to find better ways to assess differentiation and immunomodulatory potential of MSCs for cell therapy. Some studies have examined MSC shape and in vitro motility and correlated them with senescence and intrinsic differentiation capacity [43, 44]. Herein, we have identified SIRT3 as a potential point of intervention to enhance the in vitro expansion of MSCs for clinical utilization.

Stem cell aging is hypothesized to be due to cellular and genomic damages, causing the cell to arrest and eventually undergo apoptosis or senescence. The self-renewal potential and differentiation capacity of stem cells become dysregulated with age [29, 32]. Because SIRT3 generally decreases with age, this may provide one explanation as to why MSCs from older donors are less efficacious for in vitro immunopotency assays and for clinical utilization. Furthermore, it may also explain why in vitro expansion of MSCs also reduces these properties. A major source of cellular damage is conferred by ROS, natural by-products of cellular respiration [45], and ROS levels in stem cells increase dramatically with age [46]. Despite what we know about ROS in stem cell aging, it remains unclear how ROS levels increase with age in stem cells and whether or not this is reversible. Most ROS are produced by the mitochondria. SIRT3 is the major protein deacetylase in the mitochondria, where its actions decrease ROS. Additionally, SIRT3 initiates metabolic reprogramming toward more efficient electron transport and fuel usage away from carbohydrate catabolism, which is thought to result in reduced ROS production.

These findings are consistent with two other reports of SIRT3 in stem cells. Firstly, in myoblasts, SIRT3 expression peaks right before differentiation, and depletion of SIRT3 impairs differentiation [47, 48]; likewise, we found that depletion of SIRT3 impaired MSC differentiation. Secondly, in HSCs, SIRT3 has been shown to slow age-associated decline of function and senescence, and overexpression in HSCs from older donors enhances their longevity and differentiation [31]; similarly, we found that overexpression of SIRT3 in MSCs reduced senescence and oxidative stress. Therefore, it appears that SIRT3 is profoundly important for the maintenance and longevity of many types of stem cells.

With regard to differentiation capacity, studies have shown conflicting results. Some studies suggest that differentiation capacity decreases in general with age [23], while other studies suggest that adipogenic differentiation increases while osteogenic differentiation decreases with age [49, 50]. This latter explanation, the so-called “adipogenic switch,” may serve as a mechanism by which osteogenesis decreases and adipogenesis increases as we age. This is in line with the finding that loss of SIRT3 reduces the differentiation capacities of MSCs.

## 6. Conclusions

SIRT3 plays a crucial role in MSC biology. SIRT3 is required for MSC differentiation. SIRT3 decreases with age, and overexpression of SIRT3 in aged MSCs improves longevity and differentiation capacity. These results suggest modulating SIRT3 expression to enhance ex vivo expansion of MSCs to improve their clinical utilization.

## Supplementary Material

Supplemental Table 1: Primer sequences for qRT-PCR.
Supplemental Figure 1: Sirtuin family gene expression in MSCs after passaging.
Supplemental Figure 2: Sirtuin family gene expression in MSCs after differentiation into adipocytes and osteoblasts.

## Abbreviations

DHE: Dihydroethidium
HSC: Hematopoietic stem cell
MSC: Mesenchymal stromal/stem cell
NAC: N-Acetyl cysteine
ROS: Reactive oxygen species
SIRT: Sirtuin.
