# Supplementary material for: SIRT3 Enhances Mesenchymal Stem Cell Longevity and Differentiation
Source: Oxid Med Cell Longev. 2017 Jun 21;2017:5841716. doi: 10.1155/2017/5841716 (PMC5499245; doi:10.1155/2017/5841716)

**Supplemental Table 1: Primer sequences for qRT-PCR.**

| **Gene** | **Forward Primer (5’-3’)** | **Reverse Primer (5’-3’)** |
| --- | --- | --- |
| *18SRRNA* | GTAACCCGTTGAACCCCATT | CCATCCAATCGGTAGTAGCG |
| *GAPDH* | GGAGCGAGATCCCTCCAAAAT | GGCTGTTGTCATACTTCTCATGG |
| *ACTB* | CATGTACGTTGCTATCCAGGC | CTCCTTAATGTCACGCACGAT |
| *SIRT1* | TAGCCTTGTCAGATAAGGAAGGA | TGTTCTGGGTATAGTTGCAAGT |
| *SIRT2* | TGCGGAACTTATTCTCCCAGA | GAGAGCGAAAGTCGGGGAT |
| *SIRT3* | ACCCAGTGGCATTCCAGAC | GGCTTGGGGTTGTGAAAGAAG |
| *SIRT4* | AAGATGAGCTTTGCGTTGACT | CCAATGGAGGCTTTCGAGCA |
| *SIRT5* | TCCAGCGTCCACACGAAAC | AACTTGGCCGAGCCATTTTCA |
| *SIRT6* | CCCACGGAGTCTGGACCAT | CTCTGCCAGTTTGTCCCTG |
| *SIRT7* | GACCTGGTAACGGAGCTGC | CGACCAAGTATTTGGCGTTCC |

**Supplemental Figure 1: Sirtuin family gene expression in MSCs after passaging.**

Quantitative RT-PCR demonstrating mRNA expression of all 7 sirtuins in 3 strains of MSCs passaging for up to 11 passages. Cycle threshold (C_T_) values were normalized to the combination of 3 housekeeping genes (*RRN18S, GAPDH* and *ACTB*). Relative mRNA levels were determined by calculating 2^-ΔC_T_ and normalizing to the early passage (P3) MSCs for each sirtuin. Bars represent means ± SEM for 3 strains of MSCs.

**
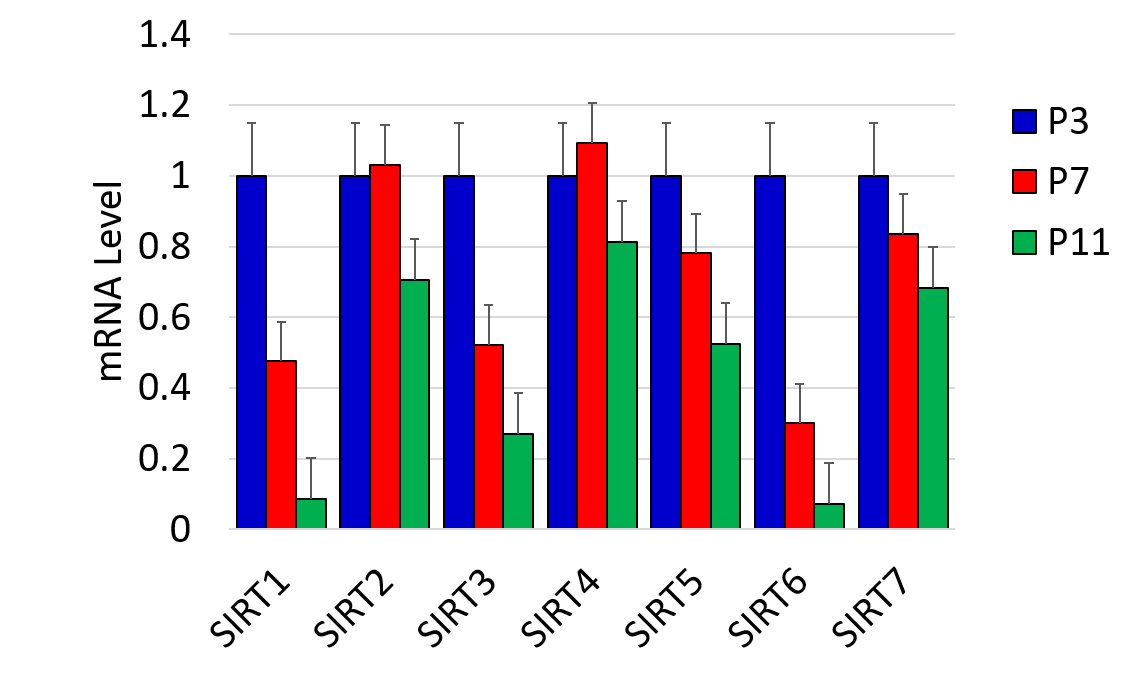
**

**Supplemental Figure 2: Sirtuin family gene expression in MSCs after differentiation into adipocytes and osteoblasts.**

Quantitative RT-PCR demonstrating mRNA expression of all 7 sirtuins in 3 strains of MSCs after 21 days of exposure to appropriate differentiation media. Bars represent means ± SEM. Cycle threshold (C_T_) values were normalized to the combination of 3 housekeeping genes (*RRN18S, GAPDH* and *ACTB*). Relative mRNA levels were determined by calculating 2^-ΔC_T,_ which were then normalized to undifferentiated MSCs for each sirtuin. Bars represent means ± SEM for 3 strains of MSCs.


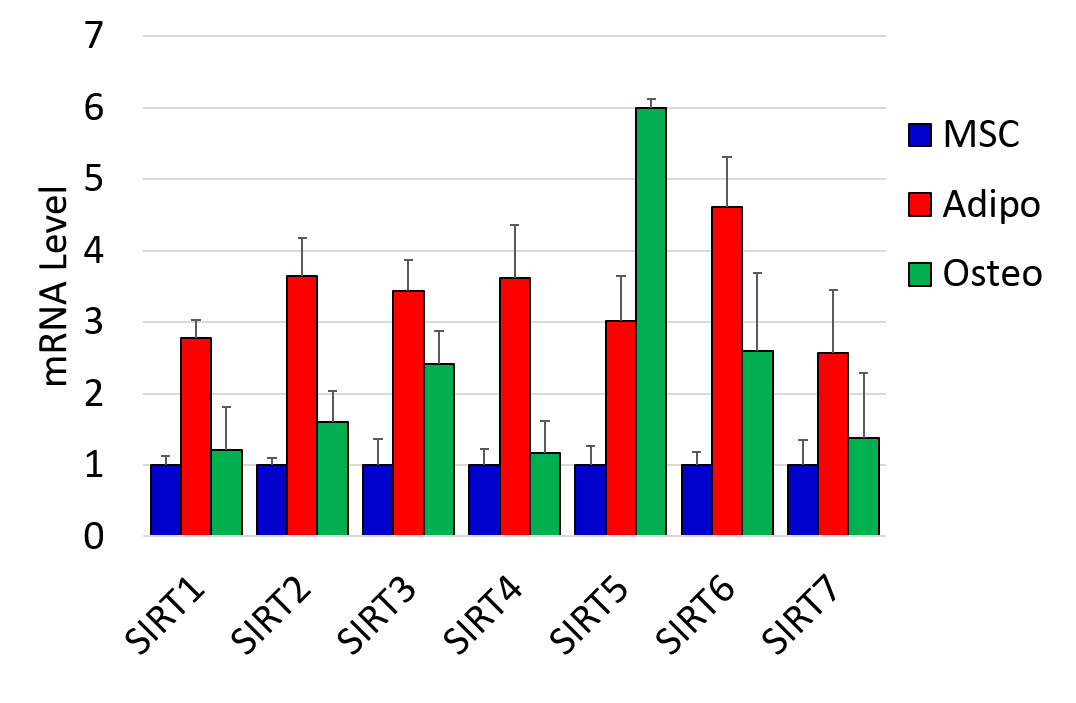

Supplement: Supplementary file 1 — Supplemental Table 1: Primer sequences for qRT-PCR. Supplemental Figure 1: Sirtuin family gene expression in MSCs after passaging. Supplemental Figure 2: Sirtuin family gene expression in MSCs after differentiation into adipocytes and osteoblasts. [file 5841716.f1.docx]
