# Supplementary material for: SIRT3 Enhances Mesenchymal Stem Cell Longevity and Differentiation
Source: Oxid Med Cell Longev. 2017 Jun 21;2017:5841716. doi: 10.1155/2017/5841716 (PMC5499245; doi:10.1155/2017/5841716)
Supplement: Supplementary file 2 [file 5841716.f2.docx]

**Supplemental Figure 1: Sirtuin family gene expression in MSCs after passaging.**

Quantitative RT-PCR demonstrating mRNA expression of all 7 sirtuins in 3 strains of MSCs passaging for up to 11 passages. Cycle threshold (C_T_) values were normalized to the combination of 3 housekeeping genes (*RRN18S, GAPDH* and *ACTB*). Relative mRNA levels were determined by calculating 2^-ΔC_T_ and normalizing to the early passage (P3) MSCs for each sirtuin. Bars represent means ± SEM for 3 strains of MSCs.

**
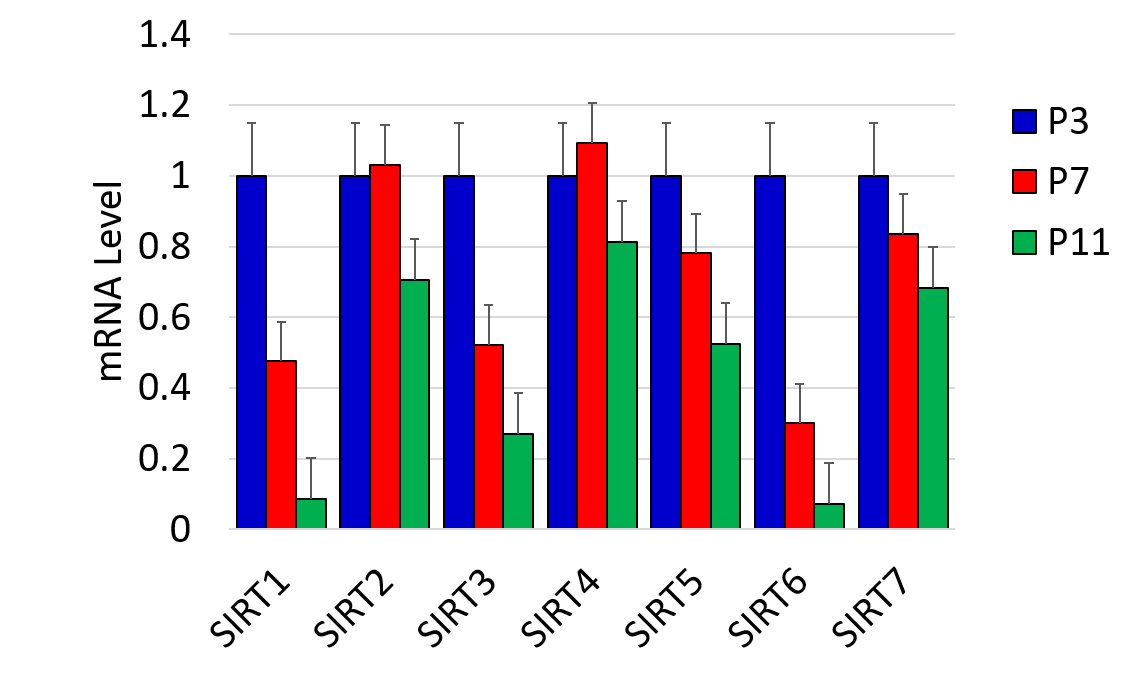
**
