# Supplementary material for: SIRT3 Enhances Mesenchymal Stem Cell Longevity and Differentiation
Source: Oxid Med Cell Longev. 2017 Jun 21;2017:5841716. doi: 10.1155/2017/5841716 (PMC5499245; doi:10.1155/2017/5841716)

**Supplemental Figure 2: Sirtuin family gene expression in MSCs after differentiation into adipocytes and osteoblasts.**

Quantitative RT-PCR demonstrating mRNA expression of all 7 sirtuins in 3 strains of MSCs after 21 days of exposure to appropriate differentiation media. Bars represent means ± SEM. Cycle threshold (C_T_) values were normalized to the combination of 3 housekeeping genes (*RRN18S, GAPDH* and *ACTB*). Relative mRNA levels were determined by calculating 2^-ΔC_T,_ which were then normalized to undifferentiated MSCs for each sirtuin. Bars represent means ± SEM for 3 strains of MSCs.


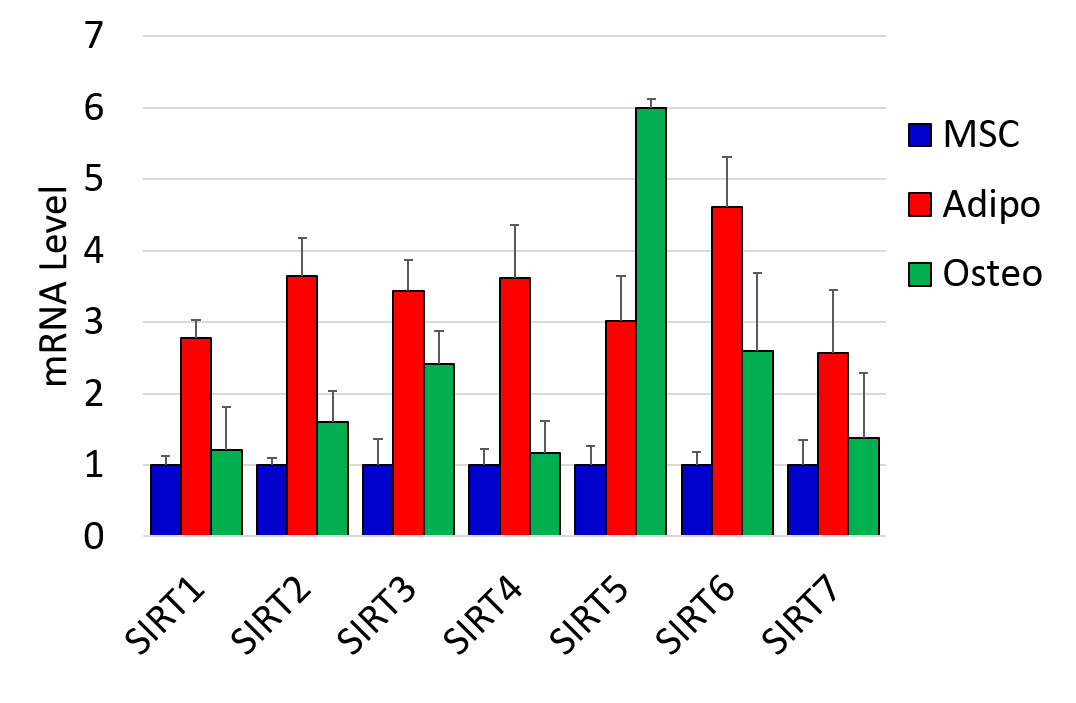

Supplement: Supplementary file 3 [file 5841716.f3.docx]
